# Squamous cell carcinoma arising in a Zenker diverticulum

**DOI:** 10.1093/jscr/rjac458

**Published:** 2022-09-28

**Authors:** Francesca Polit, Hisham F Bahmad, Jessica Wahi, Arunima Deb, Kevin Newsome, Lydia Howard, Robert Poppiti, Kfir Ben-David, Sarah Alghamdi

**Affiliations:** Arkadi M. Rywlin M.D. Department of Pathology and Laboratory Medicine, Mount Sinai Medical Center, Miami Beach, FL, USA; Arkadi M. Rywlin M.D. Department of Pathology and Laboratory Medicine, Mount Sinai Medical Center, Miami Beach, FL, USA; Department of General Surgery, Mount Sinai Medical Center, Miami Beach, FL, USA; Arkadi M. Rywlin M.D. Department of Pathology and Laboratory Medicine, Mount Sinai Medical Center, Miami Beach, FL, USA; Department of Translational Medicine, Herbert Wertheim College of Medicine, Florida International University, Miami, FL, USA; Arkadi M. Rywlin M.D. Department of Pathology and Laboratory Medicine, Mount Sinai Medical Center, Miami Beach, FL, USA; Department of Translational Medicine, Herbert Wertheim College of Medicine, Florida International University, Miami, FL, USA; Arkadi M. Rywlin M.D. Department of Pathology and Laboratory Medicine, Mount Sinai Medical Center, Miami Beach, FL, USA; Department of Translational Medicine, Herbert Wertheim College of Medicine, Florida International University, Miami, FL, USA; Department of General Surgery, Mount Sinai Medical Center, Miami Beach, FL, USA; Department of Translational Medicine, Herbert Wertheim College of Medicine, Florida International University, Miami, FL, USA; Arkadi M. Rywlin M.D. Department of Pathology and Laboratory Medicine, Mount Sinai Medical Center, Miami Beach, FL, USA; Department of Translational Medicine, Herbert Wertheim College of Medicine, Florida International University, Miami, FL, USA

## Abstract

Squamous cell carcinoma (SCC) arising in a Zenker diverticulum (ZD) is an extremely rare entity. Approximately 50 cases have been reported worldwide. We report a case of a 74-year-old man who presented to our institution with chronic regurgitation, dysphagia and halitosis. The patient was initially seen in 2015 at which point he reported a 10-year history of these symptoms and was diagnosed with ZD. A barium swallow was done revealing a large posterior esophageal diverticulum with significant residual contrast within the diverticulum lumen. Given these findings, he was taken for open surgical excision where a SCC was identified. Although it is extremely rare for a SCC to occur in a ZD, patients with ZD must undergo regular surveillance endoscopy of the esophagus and the diverticulum itself to identify any suspicious mass or lesion arising in within.

## INTRODUCTION

Squamous cell carcinoma (SCC) arising in a Zenker diverticulum (ZD) is an extremely rare entity. Approximately 50 cases have been reported worldwide [1]. The incidence of SCC arising in a ZD ranges from 0.3 to 7% [2, 3]. Potential complications of ZD include ulceration, perforation and recurrent aspiration pneumonia, all of which can be only managed with surgery. Nevertheless, having a SCC arise in a ZD is a serious complication that needs to be identified early. Therefore, it has been recommended to perform a diverticulectomy in symptomatic patients to reduce the risk of the development of a Zenker carcinoma [4]. Here, we report a rare case of a 74-year-old man with a SCC arising in a ZD, treated by resection.

## CASE REPORT

**Figure 1:**
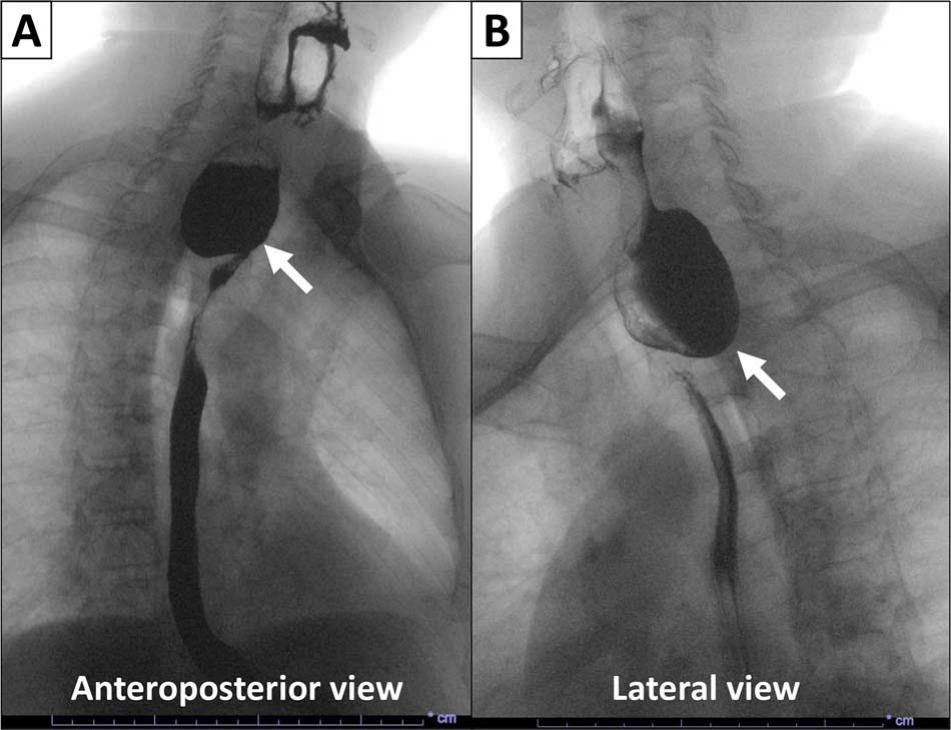
X-ray esophagogram performed in 2015. Esophagogram with barium suspension showed a 4 × 5 cm Zenker’s diverticulum with a 1.7 cm wide diverticular neck.

This is a case of a 74-year-old man with a history of diabetes mellitus and hyperlipidemia, who presented to our institution with chronic regurgitation, dysphagia and halitosis. The patient was initially seen in 2015 at which point he reported a 10-year history of these symptoms and was diagnosed with ZD (Fig. 1). He had frequent chocking and aspiration events and worsening symptoms in the past few months. Surgical history included cholecystectomy in 2010 and partial gastrectomy in 1989 for stomach cancer. Vital signs on admission were normal. His body mass index was 26.71 kg/m^2^. Physical examination and laboratory values on admission were normal. The patient never smoked and does not drink alcohol.

**Figure 2:**
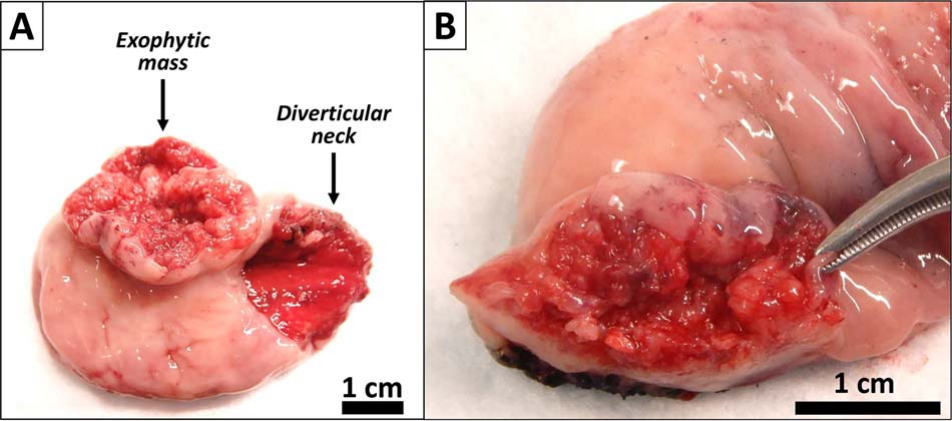
Gross images of the diverticulectomy specimen. (**A**) Exophytic and ulcerated mass identified within the Zenker’s diverticulum. (**B**) Sectioning across the mass showed a white cut surface involving the full thickness of the esophageal wall and grossly abutting the serosal surface.

Pre-operative computed tomography imaging demonstrated a 5.0 × 4.4 × 2.4 cm diverticulum off of the proximal posterior esophagus, consistent with a ZD. In addition, a barium swallow revealed a large posterior esophageal diverticulum with significant residual contrast within the diverticulum lumen. Given these findings, the patient was taken for open surgical excision or Zenker diverticulectomy. A large exophytic and ulcerated mass was identified within the diverticulum (Fig. 2). The specimen was sent to pathology. A frozen section of the mass was obtained revealing SCC.

**Figure 3:**
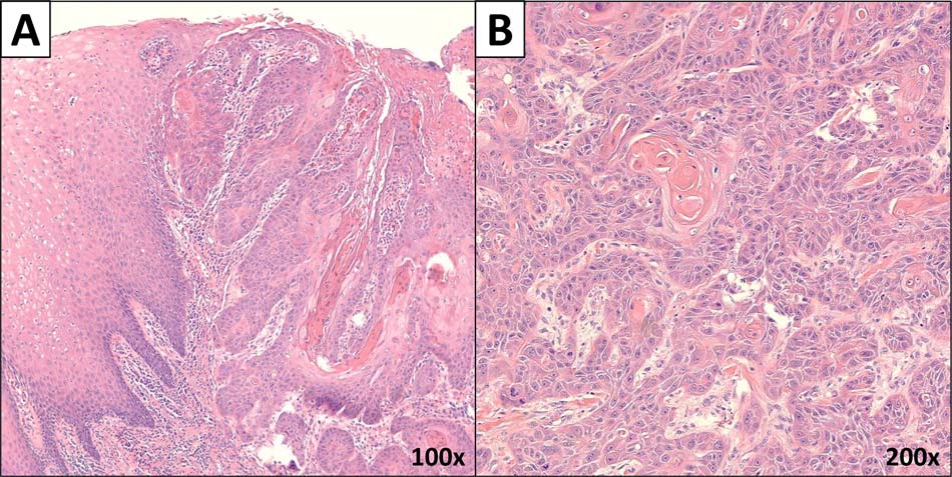
Microscopic images of the mass. (**A**) Low power microscopic image showing moderately differentiated SCC invading the submucosa (×100 magnification). (**B**) High power image of the carcinoma (×200 magnification).

**Figure 4:**
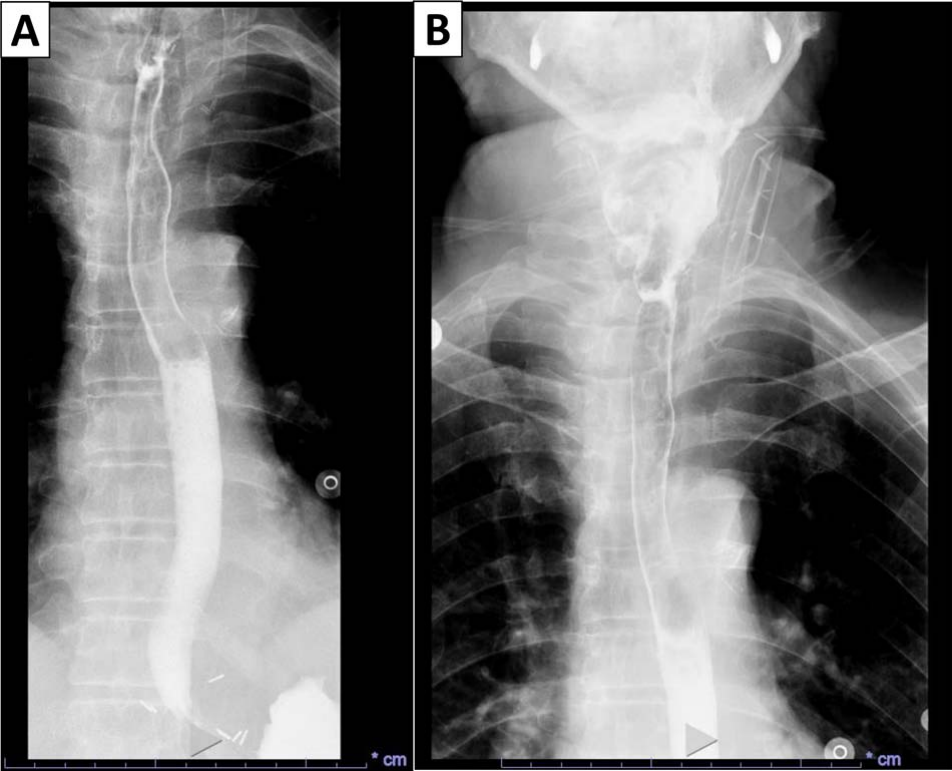
X-ray esophagogram performed postoperatively. No postoperative leak and no residual ZD were seen.

Gross examination revealed a fungating, exophytic, ulcerated, firm mass measuring 3.2 × 2.5 × 1 cm with a white cut surface involving the full thickness of the esophageal wall and grossly abutting the serosal surface. Microscopic examination showed a moderately differentiated SCC, invading the submucosa (Fig. 3). The surgical margins of resection were free of carcinoma and one cervical lymph node was identified and it showed no metastatic carcinoma. Postoperatively, the patient did well with no reported complications. An X-ray esophagogram was done showing no postoperative leak and no residual ZD (Fig. 4). He was referred to a hemato-oncologist for further management.

## DISCUSSION

In 1679, Abraham Ludlow made the first observation of a pouch that herniated through the esophagus [5]. Later, in 1877, the German pathologists Friedrich Albert von Zenker and Hugo Wilhelm von Ziemssen named this condition as ‘Zenker Diverticulum’ when they described 27 cases [6]. ZD is a pharyngoesophageal herniation of the hypopharynx through an anatomically vulnerable area called Killian’s triangle [5]. It is a false diverticulum since it does not involve the muscular layer of the hypopharynx. It can develop by two distinct mechanisms, either from pull from the outside (traction) or from getting pushed from the inside (pulsion) [5]. Barium swallow combined with continuous videofluoroscopy is the standard method for diagnosing ZD. This exam provides information about the size, shape and location of the diverticulum [7]. A transcutaneous ultrasound is an alternative method for patients with trouble swallowing or risk of aspiration [8].

Risk factors for developing ZD include advanced age and male gender [5]. The prevalence of ZD ranges between 0.01 and 0.11% [9]. Small lesions (<1 cm) may not be noticeable; however, once the defect reaches a size big enough to retain food and other materials such as medications, this will drive the patients to seek medical care for dysphagia, odynophagia, halitosis, regurgitation of undigested foods, unexplained weight loss, and rarely a palpable and visible mass in the neck [1, 5, 9, 10]. Complications of ZD include aspiration pneumonia, ulceration and bleeding, fistula formation between the diverticulum and adjacent organs, vocal cord paralysis and in very rare cases SCC. Risk factors for this malignancy arising in ZD are similar to the risk factors for developing ZD, such as male gender, older age, long standing history and untreated ZD, and a larger pouch size [11]. In our case, risk factors for developing SCC include old age, male gender, long standing history of ZD and larger size. The sudden change in severity and frequency of the symptoms is a warning sign for malignant transformation, which complies with our case.

The first case of SCC arising in ZD was described by Halstead *et al*. in 1904 [12]. To make a definitive diagnosis, surgical excision and microscopic examination are needed. High clinical suspicion is paramount to detect a malignant transformation. In our case, the patient had a history of over 6 years of ZD, and suddenly the severity and frequency of his symptoms worsened. The definitive treatment for ZD is excision. Smaller defects may be treated conservatively and assessed periodically to determine growth, velocity of growth and if the patient exhibits other concerning symptoms such as weight loss and aspiration. After surgery, patients can be evaluated for residual disease with an esophagogram. In our case, the imaging that was done after the surgery demonstrated no acute leak and no residual ZD.

Treatment is aimed at symptomatic relieve. Surgical excision either by open surgical or endoscopic techniques is readily available. Nowadays endoscopic staples diverticulectomy is the preferred approach. Ultimately, treatment choice should be driven by the patient’s conditions including diverticula size and comorbidities; and the surgeon’s expertise and preference [9].

In conclusion, SCC arising in ZD is a very rare entitiy. High clinical suspicion is important to rule out a malignant transformation. Larger and more complicated diverticula should be excised. Definitive diagnosis is made by microscopic examination. Once excised, after simple diverticulectomy and clear margins, patients are expected to remain disease free. If recurrence were to happen, that would require further evaluation for malignancy.
